# Relationship between hepatitis C and kidney stone in US females: Results from the National Health and Nutrition Examination Survey in 2007–2018

**DOI:** 10.3389/fpubh.2022.940905

**Published:** 2022-08-05

**Authors:** Yang Chen, Xudong Shen, Hu Liang, Guoxiang Li, Kexing Han, Chaozhao Liang, Zongyao Hao

**Affiliations:** ^1^Department of Urology, The First Affiliated Hospital of Anhui Medical University, Hefei, China; ^2^Institute of Urology, Anhui Medical University, Hefei, China; ^3^Anhui Key Laboratory of Genitourinary Diseases, Anhui Medical University, Hefei, China; ^4^Department of Infectious Diseases, The First Affiliated Hospital of Anhui Medical University, Hefei, China

**Keywords:** kidney stone, hepatitis C virus (HCV), National Health and Nutrition Examination Survey(NHANES), nephrolithiasis, cross-section study

## Abstract

**Background:**

The main objective of this study is to explore the effects of hepatitis C (HCV) on the prevalence rate of kidney stones in US women.

**Method:**

Dates for HCV infection and kidney stones were collected from National Health and Nutrition Examination Survey (NHANES) database, a cross-sectional study. The analysis samples included adults aged ≥20 years and women from six consecutive cycles of the NHANES 2007–2018. The association between HCV infection and kidney stones was performed by using logistic regression models. Subgroup analyses were conducted to find sensitive crowds.

**Results:**

A total of 13,262 participants were enrolled, including 201 infected with HCV. After adjustment for potential confounders, we revealed a positive relationship between HCV and kidney stones (OR = 1.70, 95%CI:1.13–2.56). The crowds' statistically significant difference was characterized by other races (OR = 8.17, 95%CI:1.62–41.22) and BMI within 25–29.9 kg/m2 (OR = 2.45, 95%CI:1.24–4.83).

**Conclusions:**

HCV infection may affect the prevalence of urolithiasis in US women, even the causal relationship remains unclear, the relation deserves special attention. We considered such a study an ideal way to begin exploring the effects of HCV on kidney stones.

## Introduction

When it comes to the urinary system, nephrolithiasis (NL), also known as kidney stones, is a prevalent problem in the study field, with high rates of occurrence and recurrence (1–3). The morbidity of patients with nephrolithiasis is increasing as a result of global warming, including a fast-paced lifestyle and an unhealthy or irregular diet, among other factors (4). Kidney stones have become increasingly widespread all around the globe over the years, regardless of gender, race, or age of the patient. Note that the rate of kidney stone production varies significantly across different geographical locations and nations. The prevalence of kidney stones in the United States (5) was more than 10%, according to a recent multi-country survey study, although the rates were 9 percent and 5.8 percent in Europe (6) and China (7), respectively, according to the same study. We also found that gender plays a very important role in the epidemiology of renal calculi. According to a 2020 NHANES-based survey study (5), it was found that the prevalence of kidney stones increased significantly in women during 2008–2018, while it did not increase significantly in men. In another retrospective study that surveyed 500,000 Navy personnel, it was found that the prevalence of kidney stones was way higher in women than in men (8). To treat it, minimally invasive endoscopic procedures, such as percutaneous nephrolithotomy, flexible ureteroscopy lithotripsy, and other endoscopic procedures, are routinely performed. Although therapy has been completed, patients still have a substantial risk of recurrence of their condition (9). If it is not treated effectively, it may progress to serious consequences, such as irreversible kidney damage and end-stage renal disease. Nephrolithiasis has grown in importance as a public health concern, as well as a significant financial burden on medical healthcare systems (10).

Hepatitis C virus (HCV) is a single-stranded RNA virus with an envelope that belongs to the Flaviviridae viral family (11). It is responsible for the transmission of the disease. HCV infection is a serious public health hazard in many parts of the globe, including the United States. Worldwide, around 130–150 million individuals are estimated to be chronically infected with HCV (12), according to the WHO. HCV infection is diagnosed with the use of anti-HCV antibodies and HCV RNA (13). It is one of the most significant contributors to liver fibrosis, organ failure, and even liver cancer (14). Not only that but there is also a gender gap in HCV infection rates. HCV surveillance data typically show lower HCV detection rates in women than in men (15). However, a recent survey of HCV prevalence among drug users found a higher prevalence among women drug users (15). A Pakistani study also found significantly higher rates of HCV infection in women than in men (16). Recent investigations have shown that HCV may infect macrophage cells, which may explain the extrahepatic symptoms. Chronic HCV infection has the potential to harm other organs, including the lung (17), heart (18), and kidney. Almost all of the research conducted so far has focused only on the impact of HCV on renal function (19). The most typically documented kidney ailment linked with HCV is HCV-associated glomerular disease, which is characterized by the presence of mixed cryoglobulinemia. Although glomerular damage is often believed to be the cause of kidney disease, tubular injury has also been identified as a contributing factor. It has been found that individuals with HCV infection suffer from both glomerular and tubule damage, respectively (20). One of the most serious risks associated with kidney stone prevalence is tubular epithelial cell injury (2). Is there a correlation between the increased prevalence of kidney stones and HCV infection in women?

As a consequence, we undertook a large population-based cross-sectional investigation using the National Health and Nutrition Examination Survey database to determine if there is a link between HCV and the occurrence of kidney stones in the general population. Subgroup studies were conducted at the same time to identify the most vulnerable population. All of the studies aimed to provide some kind of proof for the prevention of renal illness on some level.

## Materials and methods

### Study design

Data for this study were obtained from the NHANES database based on big data mining methods and were conducted by the Centers for Disease Control and Prevention (CDC) (21, 22). NHANES is a nationally representative, cross-sectional survey meant to produce nationally representative estimates of the population's health and nutritional status. It is utilized for cross-sectional investigations. From 2007 through 2018, survey data was used six times in a row. The NHANES website (www.cdc.gov/nchs/nhanes/) has further information on the data.

### Participants

**Figure 1:**
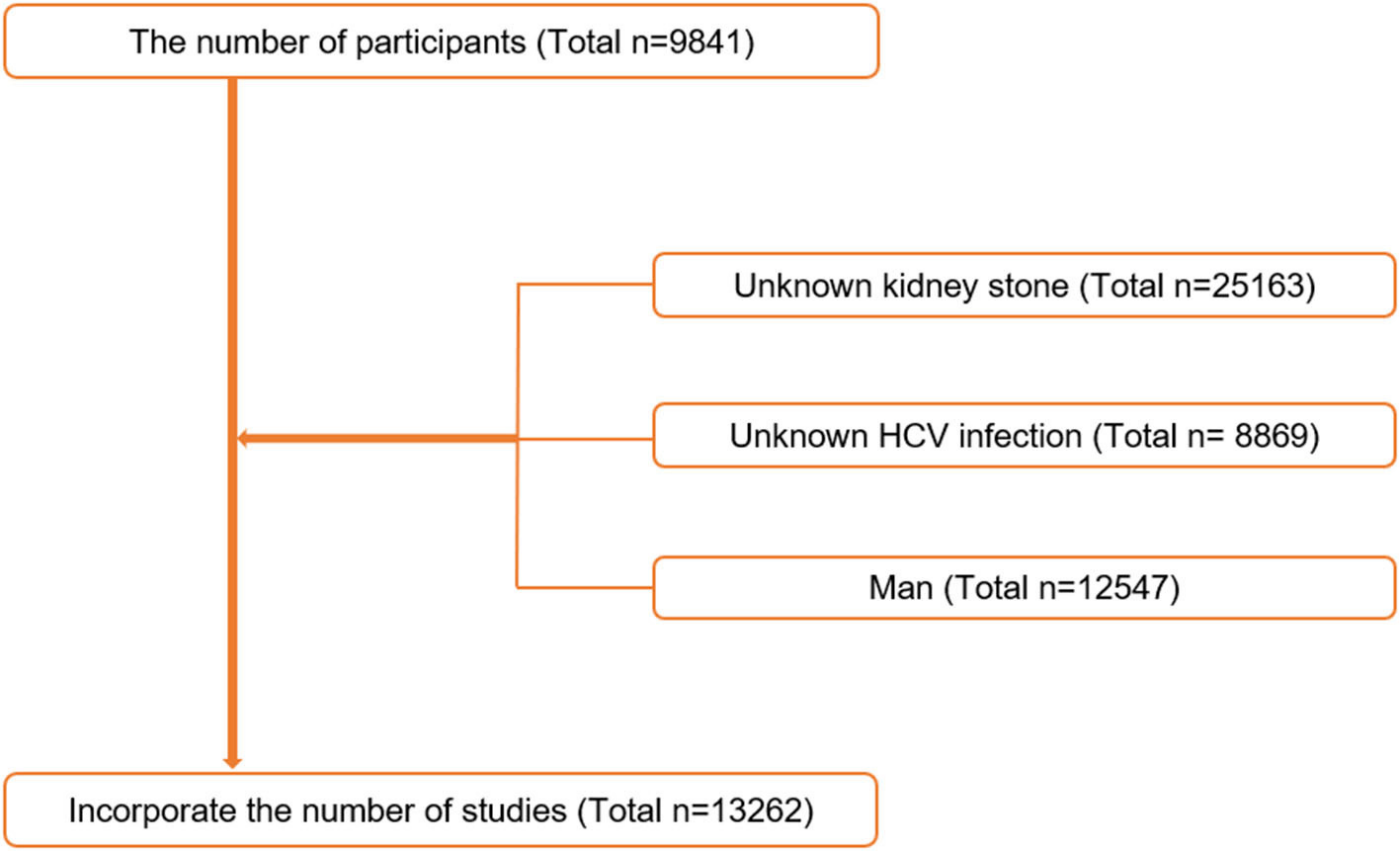
Flowchart of the sample selection from National Health and Nutrition Examination Survey (NHANES) 2007–2018.

In this research, all of the subjects were between the ages of 20 and 80. The current research has a total of 59,841 participants. The following were the criteria for exclusion: (1) unknown kidney stone (*n* = 25,163); (2) undetermined HCV infection status (*n* = 8,869); and (3) men (*n* = 12,547) (Figure 1). A total of 13,262 participants were included in the final analysis. The Institutional Review Committee of the National Center for Health Statistics(NCHS) gave its approval to the procedure.

### Variables

To identify kidney stones, the results from the KIQ026 (Do you have kidney stones?) questionnaire were used. To confirm the diagnosis of HCV infection, laboratory tests were completed. The methodologies for detecting HCV-Ab and HCV-RNA were developed from the laboratory data column's “Description of Laboratory Methodology.” HCV-Ab(+)or HCV-RNA(+) were used to define positive HCV infection. Age, race, education level, marital status, PIR (Ratio of Family Income to Poverty), physical activity, total water consumption, high blood pressure, diabetes, and alcohol users were all included in the questionnaires. The laboratory test yielded the BMI.

### Statistical analysis

Regarding the selection of weights, the principle of the official guidelines provided by NHANES is to first specify the variable that examines the smallest population and then proceed with selecting the weights corresponding to that variable. In this study, our data included MEC examination data, and according to the recommendations of the weight selection guidelines, we selected the sub-weights corresponding to MEC. According to the NHANES analysis guidelines, the new sampling weights for the combined survey cycles were constructed by dividing the 2-year weights for each cycle by six (23).

Categorical, dichotomous, and continuous variables were used to record the data. Continuous variables were expressed as mean standard deviation (SD), and dichotomous and categorical variables were expressed as counts proportions. Weighted Chi-square tests (categorical variables) and weighted one-way analysis of variance (ANOVA) (normal distribution continuous variables) or weighted Kruskal-H Wallis's test (skewed distribution continuous variables) were used to evaluate differences in clinical characteristics between groups. When <10% of the data were missing, the missing item value was replaced by means. For variables with an additional category, missing indications were produced. Because HCV-Ab data was unavailable from 2015–2016, the infection status gap was filled by the total HCV infection prevalence from 2007 to 2018.

We employed machine learning to predict the effect of each research variable on kidney stone prevalence to assess the risk of the factors linked to kidney stone prevalence (**Figure 3**). To investigate the independent connection, three logical regression models were created in our study: (1) unadjusted; (2) adjusted for age and race; and (3) adjusted for all factors. In addition, we used stratified multivariate logistic regression to conduct subgroup analyses to find acceptable groups. Statistical analyses were carried out using R 3.5.3 (http://www.r-project.org/) and Empower Stats software (http://www.empowerstats.com), with a *P*-value of 0.05 being considered statistically significant.

## Results

**Figure 2:**
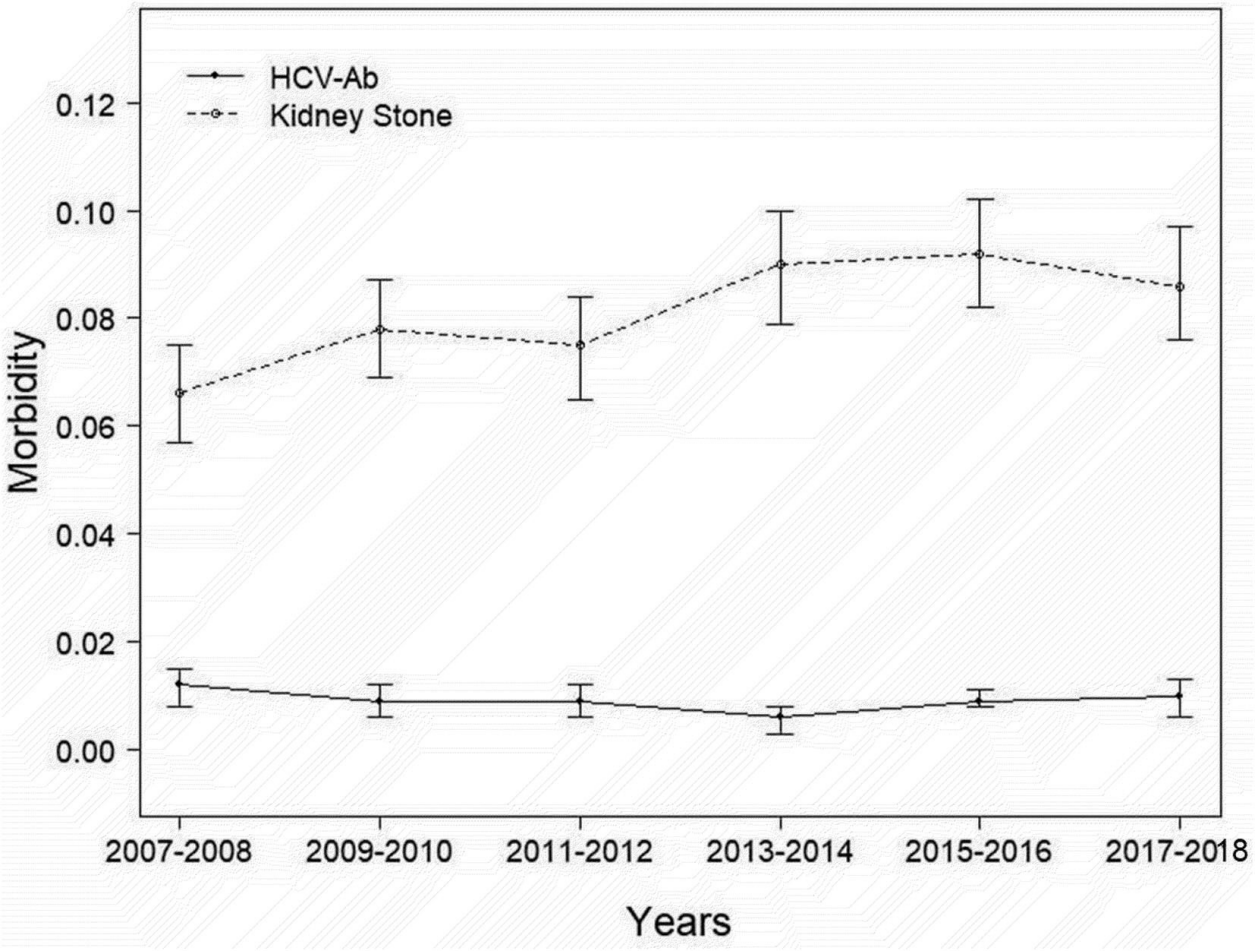
Prevalence of kidney stone and HCV infection from 2007–2018.

**Figure 3:**
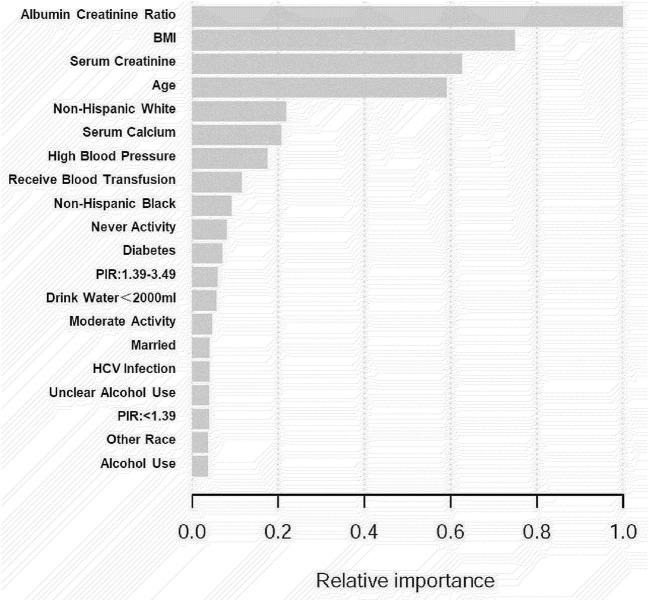
Importance of the variables in the machine learning model, scaled to a maximum of one.

A total of 13,262 people were included in our study, and the necessary variables are listed in Table 1. In general, the morbidity rate associated with kidney stone development has been increasing (*P* for trend<0.001). However, this tendency did not hold for HCV infection prevalence (*P* for trend >0.05). In comparison to the nonstone former group, the stone former group had a greater prevalence of HCV infection (*p* < 0.0001). The results are depicted in Figure 2, as well as in Supplementary Tables 1, 2. Additionally, we applied machine learning (24) to predict the effect of each research variable on the production of kidney stones. The result indicates that HCV infection is one of the several factors influencing the production of calculi (Figure 3).

**Table 1:** Baseline characteristics of US female participants in NHANES from 2007–2018, weighted.

**Characteristic**	**Nonstone formers *N* = 12,197**	**Stone formers *N* = 1,065**	***P*-value**
Age(years)	47.69 ± 17.10	51.61 ± 15.81	<0.0001
Total calcium(mg/dl)	9.37 ± 0.37	9.38 ± 0.45	0.214
Serum creatinine(mg/dl)	0.77 ± 0.27	0.84 ± 0.66	<0.0001
BMI(kg/m^2^)	28.99 ± 7.48	31.00 ± 7.85	<0.0001
**Race(%)**			<0.0001
Mexican American	7.97	6.82	
Other hispanic	6.03	4.5	
Non-hispanic white	66.38	75.87	
Non-hispanic black	11.78	6.96	
Other race	7.85	5.84	
**Infection status(%)**			<0.0001
No	98.81	97.1	
Yes	1.19	2.9	
**Blood pressure(%)**			<0.0001
Yes	69.53	52.13	
No	30.38	47.74	
Unclear	0.09	0.14	
Education level(%)			0.4675
Less than high school	15.56	16.85	
High school	22.06	23.05	
More than high school	62.3	59.99	
Unclear	0.08	0.11	
**Marital status(%)**			0.6637
Cohabitation	60.16	61.51	
Solitude	39.8	38.46	
Unclear	0.04	0.03	
PIR(%)			0.0717
<1.39	31	34.27	
1.39–3.49	23.54	23.23	
≥3.49	38.03	34.63	
Unclear	7.43	7.87	
**Ever receive blood transfusion(%)**			<0.0001
Yes	12.1	21.53	
No	86.95	77.55	
Other	0.95	0.92	
Total water(%)			0.0562
<2000 ml	51.47	52.22	
2000–2500 ml	7.82	9.81	
More than 2500 ml	26.3	25.12	
Unclear	14.41	12.86	
Physical activity(%)			0.001
Never	32.89	37.69	
Moderate	36.74	37.04	
Vigorous	30.35	25.24	
Unclear	0.02	0.03	
**Alcohol (%)**			0.0854
Yes	22.72	25.07	
No	50.2	46.6	
Unclear	27.08	28.33	
**Diabetes (%)**			<0.0001
No	89.69	79.54	
Yes	8.37	16.67	
Borderline	1.87	3.43	
Unclear	0.07	0.36	

Statistically significant, p < 0.05; Mean+SD for continuous variables. P-value was calculated by weighted linear regression model.

% for categorical variables, P-value was calculated by weighted chi-square test.

HCV, Hepatitis C virus; BMI, Body mass index (kg/m2); PIR, Ratio of family income to poverty.

The major objective of this study was to establish a causal relationship between HCV infection status and the prevalence of kidney stone development. We conducted multivariate logistic regression analysis. Three models were developed in accordance with the guidelines of the Strengthening the Reporting of Observational Studies in Epidemiology (STROBE) statement (25). In an unadjusted model, kidney stone development was associated with HCV infection in US women (OR = 1.96, 95%CI:1.31–2.92). Following that, after adjusting for age and race, we developed a second model, the pattern was similar to the first. Surprisingly, the results were substantially equal before and after adjustment for all variables (OR = 1.70, 95%CI:1.13–2.56). Subgroup analysis revealed that this negative connection trend was more evident in subgroups that comprised other races (OR=8.17, 95% CI:1.62–41.22) and with BMI between 25 and 29.9 kg/m^2^ (OR = 2.45, 95% CI:1.24–4.83) (Table 2).

**Table 2:** Analysis between confounders and renal stone prevalence.

**Characteristic**	**Model 1 OR(95% CI)**	**Model 2 OR(95% CI)**	**Model 3 OR(95% CI)**
HCV(–)	1	1	1
HCV(+)	1.96(1.31, 2.92)	1.96(1.31, 2.93)	1.70(1.13, 2.56)
**Subgroups**			
**Race**			
Mexican American	2.24(0.65, 7.73)	2.07(0.58, 7.40)	1.75(0.47, 6.54)
Other hispanic	2.36(0.68, 8.21)	1.90(0.52, 6.94)	1.92(0.51, 7.28)
Non-hispanic white	1.60(0.88, 2.91)	1.50(0.82, 2.76)	1.49(0.81, 2.75)
Non-hispanic black	1.90(0.85, 4.22)	1.49(0.65, 3.40)	1.41(0.61, 3.25)
Others	10.83(2.54, 46.13)	5.40(1.08, 26.94)	8.17(1.62, 41.22)
**BMI (kg/m** ^**2**^ **)**			
<25	2.60(1.16, 5.83)	1.95(0.84, 4.49)	2.10(0.91, 4.86)
25–30	2.53(1.31, 4.88)	1.99(1.01, 3.93)	2.45(1.24, 4.83)
>30	1.38(0.73, 2.62)	1.39(0.72, 2.67)	1.24(0.64, 2.40)

Model 1, no covariates were adjusted.

Model 2, Model 1+age, race were adjusted.

Model 3, Model 2+diabetes, blood pressure, education, marital status, BMI, serum calcium, PIR, ever receive blood transfusion, total water, physical activity, alcohol use, serum creatinine were adjusted. The subgroup analysis was stratified by race and BMI, not adjusted for the stratification variable itself.

## Discussion

Nephrolithiasis is characterized by a high prevalence, a high recurrence rate, and a varied prognosis (4, 26). Procrastinating the start of treatment can result in serious impairment of renal function. Earlier research suggested that kidney stones are a complicated chronic systemic condition (27). At the moment, the treatment regimen for renal calculi includes surgery, rehabilitation, and medication. Rapid advancements in science and technology aided in the evolution of the surgical approach from open surgery to a variety of less invasive endoscopic procedures. Despite this, current treatment for kidney stones focuses mostly on clinical symptoms, with no commonly available etiological medication. The basic cause of kidney stone production and recurrence remains unknown (28). As a result, understanding important risk factors for kidney stones is of critical clinical importance.

Our investigation indicated that the prevalence of kidney stones was increasing every 2 years, and the findings in the literature corroborated our findings (3, 5). However, because the morbidity of HCV-Ab did not follow the epidemiology of nephrolithiasis from 2007 to 2018, we believe that it may eventually come down to effective prevention and treatment measures (29). Although HCV infection is curable, due to the large number of people who are ignorant of their illness, HCV infection continues to be a significant public health problem (30).

The purpose of this initiative was to investigate the association between HCV infection and renal calculi. The NHANES database was mined and analyzed for massive, organized, population-based cross-sectional data. We discovered that the number of HCV-infected people in the kidney stone group was significantly higher than the number of HCV-infected subjects in the non-stone group. The most important finding of our current investigation is the identification of HCV virus status as a risk factor for kidney stone prevalence using multivariate logistic models. This connection persisted after correcting for all the confounding variables. According to the link between HCV(+) and nephrolithiasis, the most significant crowds were those with BMI of 25–29.9 kg/m^2^ and other races. Overweight (BMI 25–29.9 kg/m2) and obesity (BMI ≥ 30 kg/m2) are risk factors for the development of kidney stones, and Parvin (31) found a higher probability of kidney stones in the overweight and obese population in a study in 2021. The results of a cohort study on BMI and prevalence of kidney stones by Korean scholar Kim (32) in 2019 showed that in the metabolically healthy population, overweight and obesity have OR = 1.12, 95%: 13–1.22 and OR = 1.72, 95%:1.21–2.44, while in the metabolically unhealthy population, overweight and obesity have OR = 1.27, 95%. 1.20–1.34 and OR = 1.36, 95%:1.22–1.51. The results of this paper showed that in the female population with BMI of 25–29.9 kg/m2, OR = 2.45, 95%CI:1.24–4.83, suggesting that HCV infection is positively associated with the prevalence of kidney stones, and in the female population with BMI > 30 kg/m2, OR = 1.24, 95%CI:0.64–2.40, suggesting that HCV infection was positively associated with the prevalence of kidney stones, but not significantly, which may be related to the small sample size and needs to be verified in a large sample multicenter ready control trial.

Simultaneously, machine learning was utilized to determine whether certain variables have an effect on the occurrence of kidney stones in this study. Data may be ranked using machine learning according to their influence. The most relevant factors in the model of kidney stone development were the albumin creatinine ratio, BMI, serum creatinine, age, non-Hispanic white, high blood pressure, HCV infection status, and so on. Some research have been carried out to compare the advantages and disadvantages of machine learning approaches to traditional regression techniques. As of yet, however, the findings have been considerably disparate. Studies have revealed that logistic regression may be as accurate as, or, perhaps, more accurate than other machine learning algorithms in certain situations (20). Other research, on the other hand, have shown that machine learning approaches are more trustworthy than traditional regression analysis (24). Consequently, according to Wolpert's “No Free Lunch Theorem,” no single strategy will be the most accurate in every situation, thus, comparisons of strategies across various study topics and datasets may provide different findings. Both machine learning and logistic regression models in this study indicated HCV infection as a risk factor for kidney stone development, which is a strong start in this area of research.

Our research shows for the first time that HCV infection may have an effect on kidney stone development, utilizing a cross-sectional study. As a result, there has been no research on its putative mechanism. We hypothesize the following causes based on past research on its correlation: It is widely established that renal tubular epithelial cell injury is a critical stage in the processing of kidney stones prevalence. Although chronic kidney disease (CKD) is now recognized as an extrahepatic manifestation of hepatitis C virus, it is often advanced. Researchers are reporting that tubulointerstitial damage is the early stage of renal manifestation rather than glomerular damage (20), which is positive. An Italian study of 98 cirrhotic individuals found tubular involvement to be the most prevalent kidney abnormality (33). The HCV core protein has also been shown to be more prevalent in the renal tubules of patients with HCV infection (34). In the proximal renal tubular epithelial cells, activation of caspases 3, 8, and 9 by HCV has been found to impact tubular barrier function directly in renal epithelial cells, favoring apoptotic cascades (35). As a result of the damage to the renal tubular cells, crystals are readily formed on them. Crystals adhering to the surface of renal tubular cells are taken up by the cells. In due course, a stone is created from the crystals and crystal aggregates that have grown in the preceding step.

The following are some of our study's limitations: (a) Because this was a cross-sectional research, it cannot prove a causal link between HCV infection and the likelihood of kidney stone development; (b) It should be noted that the NHANES only included “self-reported” data on kidney stone history, which excluded asymptomatic stones. Another drawback is that we were unable to determine the sort of renal calculi that were present; and (c) Eating habits may have an impact on kidney stone development. However, no dietary information or characteristics related with kidney stone production were collected.

## Conclusions

Participants with HCV infection had a higher chance of acquiring kidney stones. Infection with HCV may be a risk factor for the production of kidney stones in US women.

## Data availability statement

The datasets presented in this study can be found in online repositories. The names of the repository/repositories and accession number(s) can be found in the article/Supplementary material.

## Ethics statement

The studies involving human participants were reviewed and approved by NCHS's Institutional Review Board reviewed and approved the study protocol. The patients/participants provided their written informed consent to participate in this study.

## Author contributions

YC: conceptualization, methodology, and software. XS: data curation and writing—original draft. HL and GL: visualization and investigation. KH: supervision and software. CL and ZH: writing—review and editing. All authors contributed to the article and approved the submitted version.

## Funding

This work was supported by the National Natural Science Foundation of China (82070724) and Natural Science Foundation of Anhui Province (1908085MH246).

## Conflict of interest

The authors declare that the research was conducted in the absence of any commercial or financial relationships that could be construed as a potential conflict of interest.

## Publisher's note

All claims expressed in this article are solely those of the authors and do not necessarily represent those of their affiliated organizations, or those of the publisher, the editors and the reviewers. Any product that may be evaluated in this article, or claim that may be made by its manufacturer, is not guaranteed or endorsed by the publisher.

## Abbreviations

HCV: Hepatitis C virus
NHANES: National Health and Nutrition Examination Survey
HCV-Ab: Hepatitis C virus antibody
HCV-RNA: Hepatitis C virus RibonucleicAcid
OR: Odds ratio
BMI: Body mass index
US: United States
PIR: Ratio of family income to poverty.
