# Supplementary material for: Relationship between hepatitis C and kidney stone in US females: Results from the National Health and Nutrition Examination Survey in 2007–2018
Source: Front Public Health. 2022 Aug 5;10:940905. doi: 10.3389/fpubh.2022.940905 (PMC9389117; doi:10.3389/fpubh.2022.940905)
Supplement: Supplementary file 1 [file Data_Sheet_1.docx]

Supplementary Table 1.Prevalence of kidney stone from 2007-2018

| Years | With kidney stone | Without kidney stone | Adjusted% |
| --- | --- | --- | --- |
| 2007-2008 | 198 | 2814 | 6.6(5.7,7.5) |
| 2009-2010 | 250 | 2956 | 7.8(6.9,8.7) |
| 2011-2012 | 210 | 2600 | 7.5(6.5,8.4) |
| 2013-2014 | 269 | 2735 | 9(7.9,10) |
| 2015-2016 | 273 | 2693 | 9.2(8.2,10.2) |
| 2017-2018 | 247 | 2617 | 8.6(7.6,9.7) |
| *P*-value for trend:*P*<0.001 | | | |

Supplementary Table 2.Prevalence of HCV infection from 2007-2018

| Years | With HCV | Without HCV | Adjusted% |
| --- | --- | --- | --- |
| 2007-2008 | 116 | 7280 | 1.6(1.3,1.9) |
| 2009-2010 | 107 | 7764 | 1.4(1.1,1.6) |
| 2011-2012 | 93 | 6952 | 1.3(1.1,1.6) |
| 2013-2014 | 104 | 7515 | 1.4(1.1,1.6) |
| 2015-2016 | / | / | 1.3(1.2,1.4) |
| 2017-2018 | 100 | 6574 | 1.5(1.2,1.8) |
| *P*-value for trend:*P=*0.16 | | | |
